# Hypersensitivity Pneumonitis Associated with Red-Vented Bulbul: A New Encounter of Bird Related Hypersensitivity Pneumonitis

**DOI:** 10.1155/2019/9572790

**Published:** 2019-12-09

**Authors:** W. D. N. L. Amarasinghe, R. Jayasekara, B. D. W. Jayamanne, T. D. K. Nalaka, W. A. D. L. Amarasiri, R. Punchihewa, A. Fernando

**Affiliations:** ^1^Central Chest Clinic, Colombo, Sri Lanka; ^2^Department of Public Health, Faculty of Medicine, University of Kelaniya, Sri Lanka; ^3^Department of Radiology, National Hospital of Sri Lanka, Sri Lanka; ^4^Department of Physiology, Faculty of Medicine, University of Colombo, Sri Lanka; ^5^Department of Pathology, National Hospital for Respiratory Diseases, Welisara, Sri Lanka

## Abstract

Bird related hypersensitivity pneumonitis (HP) is becoming more common than other forms of HP around the world. We present two cases of HP, associated with exposure to visiting birds which had nested within their homes in semi urban areas of Colombo, Sri Lanka. A 65-year-old female (case 1) and a 61-year-old male (case 2) presented to the chest clinic complaining of gradually progressive and persistent chronic dry cough and dyspnoea during the year 2018. Both were found to have close contact with red-vented bulbuls (Konda kurulla) in their homes for more than 6 months prior to onset of symptoms and denied any other risk exposures in detail history taking. In both patients, high-resolution computed tomography chest (HRCT) showed centrilobular nodules of ground glass density with significant lobular air trapping. Video-assisted thoracoscopic (VATs) lung biopsy of case 1 showed patchy and focal interstitial thickening with lymphocytic infiltrate, minimal fibrosis, and few noncaseating granulomata within the interstitium. Transbronchial lung biopsy of case 2 showed thickened alveolar septae with lympho-histiocytic infiltrate and occasional neutrophils and eosinopils. Both showed severe reduction in forced vital capacity (FVC) at presentation. Multidisciplinary diagnosis of HP associated with red-vented bulbuls was made. Both achieved good improvement in clinical, lung function, and radiological assessment following removal of offending antigen exposure and treatment with oral corticosteroids.

## 1. Introduction

Hypersensitivity pneumonitis (HP) is an immunologically-mediated inflammatory lung disease caused by repetitive inhalation of antigens in a susceptible host [1–3]. Potential causative agents are grouped as microbes, animal proteins, and chemicals [1, 4, 5]. Bird related HP is becoming the commonest form caused by high- and low-molecular-weight proteins found in feathers, faeces, and other animal products commonly of pigeons, parrots, parakeets, love birds, cockatoos, budgerigars, and fowl [4, 6–8]. We report two cases that presented to the Central Chest Clinic, Sri Lanka with a multidisciplinary diagnosis of subacute HP associated with red-vented bulbuls. There have been no previously published reports of HP associated with red-vented bulbuls or any other visiting birds. Hence, this is the first paper to study this association.

## 2. Case Presentations

Both patients were retired professionals from semiurban areas in Colombo who denied exposure to any organic or inorganic particles, which can cause HP, within the last two years. However, repeated questioning revealed that there had been close contact with red-vented bulbuls which frequented their houses. The birds nested in chandeliers hung directly above the sofas in their living rooms where these patients spent most of their leisure times. Though there had been debris falling on to the sofa as the birds moved within the nests above, they had not attempted to remove the nests as they enjoyed their presence. The living rooms had been kept closed during the periods when the birds reproduced, to protect the eggs and nestlings. Additionally, both patients had closely handled the nestlings until they fledged. This confirmed that there had been continuous exposure to antigens of red vented bulbuls for around 8–10 hours per day for 10–18 months with a proximity of 2–3 meters in a closed or partially closed environment, in these patients.

### 2.1. Case 1

**Figure 1:**
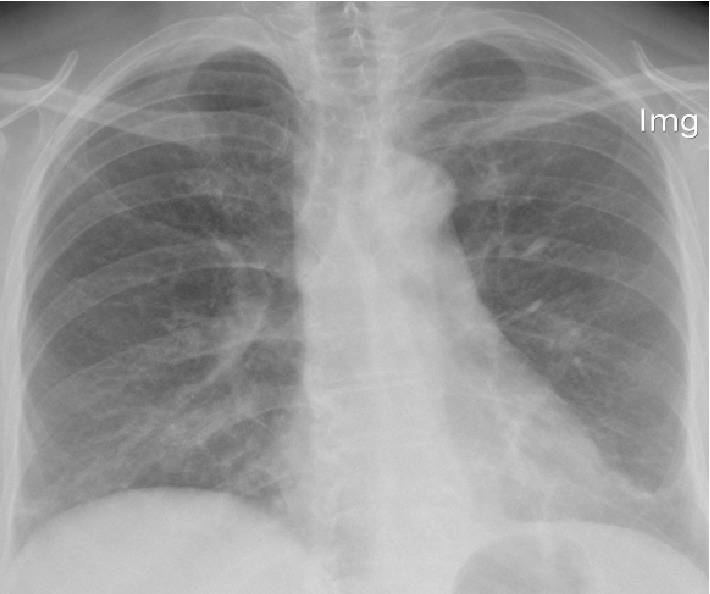
Chest X-ray shows patchy opacifications in right mid zone and reticular nodular shadows in mid zones of both lung fields.

**Figure 2:**
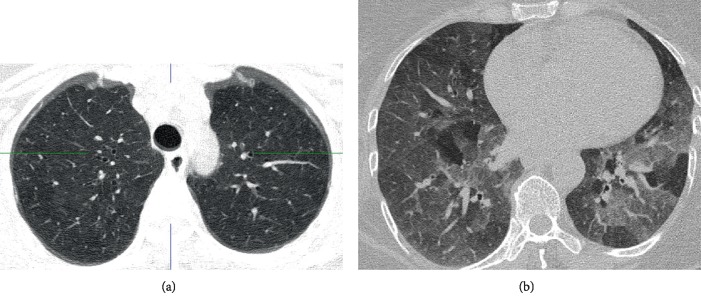
HRCT chest demonstrating upper lobe predominant centrilobular nodules of ground glass density (a). Expiratory HRCT films shows air trapping in lobules that had decreased attenuation on inspiratory film and centrilobular nodules of ground glass density (b).

**Figure 3:**
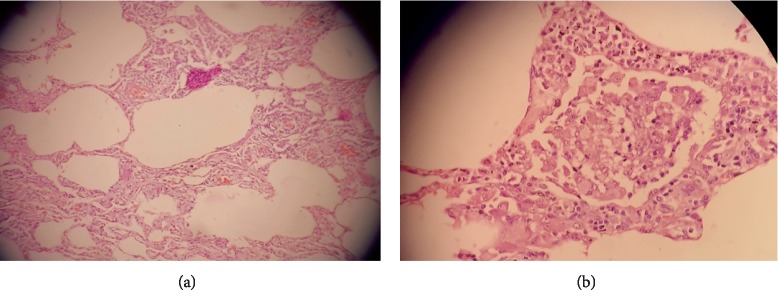
VATS biopsy shows interstitial thickening with few noncaseating granulomata (a). High power view of a noncaseating granulomata (b).

A 65-year female presented to the clinic with insidious-onset persistent and progressive dyspnoea, cough, and wheezing for six months. She was a diagnosed diabetic and had a history of allergic rhinitis. She revealed close contact with red-vented bulbul birds for around one-and-half years. Erythrocyte sedimentation rate (ESR) was 41 mm/h. Chest radiography showed patchy opacifications in right mid zone and reticular nodular shadows in mid zones of both lung fields (Figure 1). Saturation at rest was 95% and there was 7% desaturation during the 6-minute walk test where she walked a distance of 480 meters. Spirometry and body plethysmography showed severe restriction and air trapping (FVC 49.5%, total lung capacity-TLC-76%, residual volume-RV-110%, RV/TLC-158%), with reduction in diffusing capacity with patchy parenchymal involvement (carbon monoxide diffusing capacity-DLCO-67%, carbon monoxide transfer coefficient-KCO-99%). HRCT showed centrilobular nodules of ground glass density in upper zones, basal ground glass opacities, and significant lobular air trapping (Figure 2). Video-assisted thoracoscopic lung biopsy showed patchy and focal interstitial thickening with lymphocytic infiltrate, minimal fibrosis, and several noncaseating granulomata within the interstitium (Figure 3).

### 2.2. Case 2

**Figure 4:**
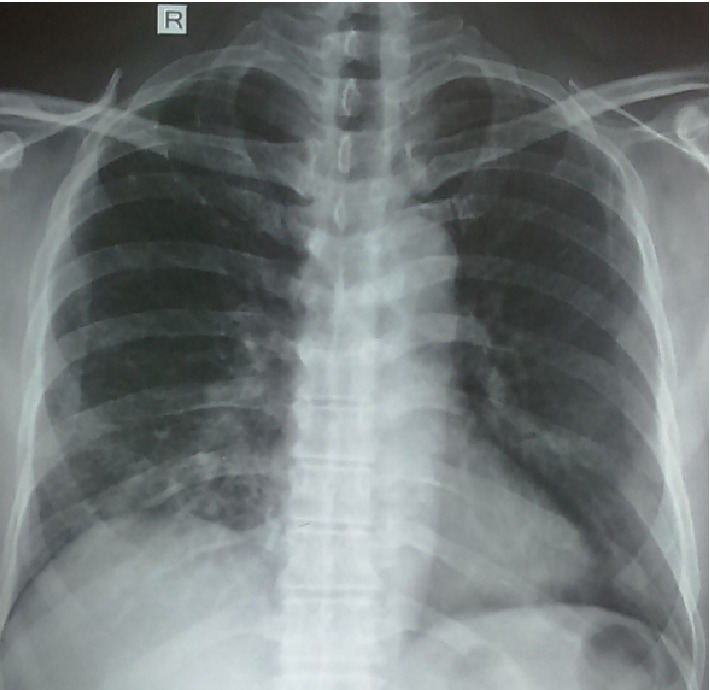
CXR- Patchy opacifications in right mid zone and reticular nodular shadows in mid zones of both lung fields.

**Figure 5:**
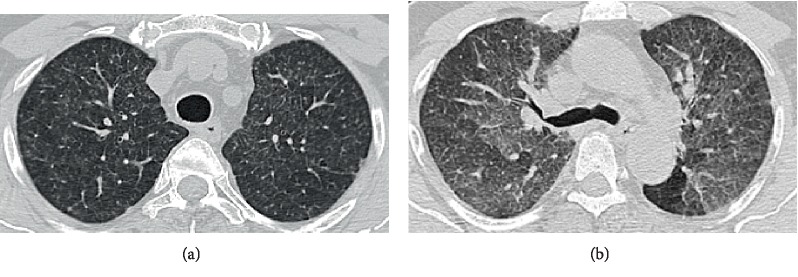
HRCT demonstrating upper lobe predominant centrilobular nodules of ground glass density (a). Expiratory HRCT slices show air trapping in lobules that had decreased attenuation on inspiratory slices with centrilobular nodules of ground glass density (b).

A 61-year-old male presented with insidious-onset, persistent and progressive dyspnoea and dry cough for four months. At the onset, he had low grade fever and constitutional symptom which subsided over a few weeks. He was a known hypertensive. He revealed close contact with red-vented bulbuls for around ten months. ESR was 78 mm/h. Chest radiography showed reticular nodular shadows in mid and lower zones of both lung fields (Figure 4). Spirometry showed severe restriction (FVC 52.8%, TLC-56%, RV-65%) with a normal diffusing capacity (DLCO-86%, KCO-180%). HRCT showed centrilobular nodules of ground glass density in all three zones and significant lobular air trapping (Figure 5). Bronchial wash cytology revealed inflammatory cells (249/cumm) with predominant lymphocytes (80%). Transbronchial lung biopsy showed thickened alveolar septae with lympho-histiocytic infiltrate and occasional neutrophils and eosinophils. Some of the alveolar spaces contained foamy histiocytes. Fibrosis was not evident.

A diagnosis of subacute HP associated with exposure to red vented bulbuls was made for both cases at a multidisciplinary meeting for interstitial lung diseases held at the National hospital of Sri Lanka. The patient of case 1 achieved good clinical and lung function improvement (FVC 91.9%) by about two months and that of case 2 showed clinical and lung function improvement (FVC 72%) around the third month of treatment with oral prednisolone (0.5 mg/kg) tail down regimen and the avoidance of the offending exposure. Both patients did not have any recurrences following avoidance of exposure to red-vented bulbuls and achieved normal FVC values in spirometry assessment with treatment of oral prednisolone within one-year follow-up period.

## 3. Discussion

Bird related HP (Bird fancier's lung) is increasingly becoming prevalent around the world and shows worse outcome than other forms [8, 9]. Bird related HP can be caused by high- and low-molecular-weight proteins (<5 *μ*m) found in feathers, faeces, and other animal products [10].

Suspicion of an association between symptoms and contact with a provoking antigen is the first step in the diagnostic process of HP, combined with the measurement of serological markers and specific IgG antibody levels, radiological findings, lung function assessment, bronchoalveolar lavage, and lung biopsy for the complete workup [4, 12]. The identification of causal antigen is impossible in about 30–60% of cases [1]. Species specific antibody are commercially available for pigeons, parrots, parakeets, cockatoos, and multiple domestic poultry species at present, and there are emerging avian associations as in this report, to be experimented [1, 11, 13]. However, laboratory measures of exposure, such as precipitin tests, specific inhalation challenge, and lymphocyte proliferation tests have failed to achieve consensus among international experts as important in diagnosing HP according to international modified Delphi survey, possibly due to the limited information on their test characteristics, lack of standardization, or limited availability [14].

Air space involvement of HP in the lung parenchyma presents as patchy ground glass opacities and/or centrilobular nodules in HRCT [15]. Shunting of blood away from poorly ventilated regions manifests as mosaic attenuation. Persistence of hypoattenuated areas in expiratory CT films indicates air-trapping [8]. Spirometry may demonstrate a restrictive lung disease pattern, with moderate to severe reduction of FVC and DLCO [10]. Lung biopsy may show cellular bronchiolitis, diffuse lymphocytic interstitial infiltration, and noncaseating granulomas [10, 16].

Proper history plays a key role in HP as early avoidance of causative associations invariably brings complete or partial recovery whereas delay would cause irreversible lung fibrosis. Multidisciplinary approach is highly recommended when the diagnosis is challenging [6].

The red-vented bulbul (*Pycnonotus cafer*) also known as Kondakurulla in Sinhala, is a resident breeder across the Indian subcontinent including Sri Lanka, and has established itself in several Pacific islands, parts of the United Arab Emirates, United States, and possibly New Zealand, not limiting to a part of the world [17–19]. They build nests in bushes at a height of around 2-3 meters and opportunistically invade houses specially using lamp shades and chandeliers for nesting, leaving an invariable risk exposure to the household [20, 21].

To our knowledge, all published cases of bird related HP are associated with birds raised as pets or for farming. An important fact to be highlighted is that when a clinician questions regarding risk exposure to birds, the patient would give a negative answer initially if they have not raised birds purposefully. The opportunity to remove the exposure would be missed in such instances. Hence, the present observation highlights the importance of specific questioning regarding this kind of casual exposures to visiting birds within living places.

Birds nesting and breeding inside houses is considered to bring prosperity to the household in some cultural beliefs. Hence updating public on harmful aspects of these associations would also be important.
